# Evaluating Predictive Value of Plasma Free Hemoglobin (PFH) in ECMO for COVID-19, Non-COVID-19 Pulmonary, and Cardiac Patients

**DOI:** 10.3390/medicina61050801

**Published:** 2025-04-25

**Authors:** Wasiq Rashid, Varshith Paduchuri, Joby Chandy, John Hodgson, Enrico Camporesi

**Affiliations:** 1 Morsani College of Medicine, University of South Florida, Tampa, FL 33606, USA; wasiqrashid@usf.edu (W.R.); paduchuri@usf.edu (V.P.); 2 Department of Anesthesiology and Perioperative Medicine, Morsani College of Medicine, University of South Florida, Tampa, FL 33606, USA; joby_chandy@teamhealth.com (J.C.); john_hodgson@teamhealth.com (J.H.); 3 TeamHEALTH Anesthesia, Tampa General Hospital, Tampa, FL 33606, USA

**Keywords:** ECMO veno-venous (VV), ECMO veno-arterial (VA), plasma free hemoglobin (PFH), respiratory insufficiency, COVID-19, cardiac insufficiency

## Abstract

*Background and Objectives*: Extracorporeal membrane oxygenation (ECMO) can support patients with severe cardiopulmonary failure, but it poses risks such as hemolysis, leading to complications. Plasma-free hemoglobin (PFH) is a hemolysis biomarker, with elevated levels linked to mortality. This study evaluates PFH and ECMO survival in COVID-19, non-COVID-19 pulmonary, and cardiac patients, focusing on late PFH spikes. *Materials and Methods*: We retrospectively analyzed 122 ECMO patients treated at our tertiary hospital (January 2020–December 2021). Patients were categorized by indication: post-COVID-19, non-COVID-19 pulmonary, or cardiac. We classified patients as Expired (died during ECMO or ≤30 days post-ECMO) or Survived (>30 days post-ECMO). Data included demographics, ECMO duration, and PFH values at 24 h and during the last 3 and 5 ECMO days. Groups were compared using two-tailed *t*-tests, with *p* < 0.05 indicating significance. *Results*: COVID-19 patients survived after significantly longer ECMO duration than non-COVID-19 pulmonary and cardiac patients. Expired COVID-19 patients had higher PFH values during the last 3 and 5 days of ECMO compared to survivors. Cardiac patients had the highest overall PFH levels regardless of mortality. No significant differences in PFH trends were observed between non-COVID-19 pulmonary and cardiac patients. *Conclusions*: Late PFH spikes correlated with mortality in COVID-19 patients, suggesting the utility of measuring late PFH spikes in ECMO management. Additionally, COVID-19 pulmonary patients survived when undergoing ECMO significantly longer than both groups, while VA ECMO was more prone to hemolysis. However, technical cannulation differences and frequent use of an Impella pump in cardiac patients may increase blood stress and PFH values.

## 1. Introduction

Extracorporeal membrane oxygenation (ECMO) is a complex technique for extracorporeal life support for patients that comes in two main types: Veno-Arterial ECMO (for patients with cardiopulmonary failure) and Veno-Venous ECMO (indicated in patients with respiratory failure). While ECMO can be lifesaving, one major complication is an increased level of hemolysis, as the passage of blood through the ECMO system’s artificial surfaces causes upregulation of the coagulation cascade, fibrinolytic pathway, and complement-mediated inflammatory responses [1]. Plasma-free hemoglobin (PFH) is a byproduct of hemolysis and can be a biomarker of this process. A substantial increase in PFH is associated with several systematic complications, including increased thrombin formation, renal failure, and inadequate tissue oxygenation leading to end-organ failure. PFH levels >50 mg/dL within 24 h of ECMO initiation are an independent mortality predictor among patients on ECMO support [1]. Additional research has also shown that mortality in ECMO was linked to higher peak levels of PFH in VV ECMO [2]. Other researchers found that a mean PFH greater than 11 mg/dL was linked to significant increases in mortality in patients with ARDS undergoing VV ECMO [3]. However, there is limited research evaluating the connection between later spikes in PFH and mortality.

Since the onset of the COVID-19 pandemic, ECMO support has also been increasingly utilized to treat these patients. COVID-19 can lead to the development of acute respiratory distress syndrome (ARDS), where ECMO support can provide more optimal oxygenation when conventional ventilation is ineffective. It can also be a potentially life-saving intervention. However, due to extensive inflammation induced by COVID-19, some reports indicate that ECMO patients with COVID-19 ARDS have worse outcomes than non-COVID-19 ARDS [4]. There are also concerns about whether artificial surfaces in contact with blood can induce a higher inflammatory response in patients with COVID-19 compared to those without [5]. High rates of complications are observed in COVID-19 patients receiving ECMO, with the most common being secondary infections, bleeding, acute kidney injury, and thrombotic events [6].

Due to the potential differences between COVID-19 patients and non-COVID-19 patients, it is appropriate to examine whether the predictive value of PFH observed in general ECMO populations also applies to COVID-19 patients, as limited research has been conducted in this area. Thus, this study aims to evaluate the association between PFH values and mortality in COVID-19 patients on ECMO and compare these findings with non-COVID-19 pulmonary and cardiac ECMO patients. Additionally, we aim to evaluate whether later spikes in PFH, in addition to early spikes from previous research, are also correlated with increased mortality risk.

## 2. Methods (Background)

### 2.1. ECMO

ECMO is typically reserved for patients with a very high degree of organ failure in which alternative medical interventions would be insufficient in preventing mortality. Due to complications of ECMO, the benefits of this cardiopulmonary therapy need to be weighed against the risks associated with increased hemolysis, thrombosis, and inflammation. In ECMO, venous blood is drained from the patient’s circulation, pressured through a membrane oxygenator and heat exchanger, and facilitated back to the body. The blood is then drained back into the venous circulation (Veno-Venous (VV) ECMO) or arterial circulation (Veno-Arterial (VA) ECMO).

### 2.2. VV ECMO

VV ECMO is typically indicated for patients exhibiting severe respiratory failure, but adequate heart function. For example, VV ECMO is the predominant form used in COVID-19 patients and is also common in ARDS, pneumonia, aspiration, barotrauma, and pneumonitis patients. VV ECMO can also be used as a “bridge” to lung transplantation.

Drainage for VV ECMO typically originates from the Inferior Vena Cava (IVC), and return is typically routed through the Superior Vena Cava (SVC) or Femoral Vein [7].

### 2.3. VA ECMO

As opposed to VV ECMO, VA ECMO is employed when a patient needs hemodynamic, circulatory, and respiratory support. For example, indications include myocardial infarction (MI), myocarditis, acute heart failure, and cardiac arrest.

Setup for VA ECMO typically consists of drainage from the femoral vein and return to the femoral artery (peripheral VA ECMO), or drainage from the right atrium or pulmonary artery and return to the left atrium, aorta, or left ventricle (central VA ECMO) [7].

### 2.4. Thrombosis and Hemolysis Due to ECMO

Initiating ECMO requires weighing risks vs. benefits due to the high potential for complications, such as thrombosis and hemolysis. Passage of blood through the ECMO system’s artificial surfaces causes upregulation of the coagulation cascade, fibrinolytic pathway, and complement-mediated inflammatory responses [1].

Two major independent variables are known to contribute to hemolysis during ECMO:

1. Venous Suction Pressures: Excessively high or prolonged negative suction pressures in the venous cannula can cause red blood cell (RBC) destruction. This mechanical stress leads to increased PFH levels, lactate dehydrogenase (LDH), and other markers of hemolysis.

2. Thrombus Formation: Hemolysis is closely associated with thrombus formation within the ECMO circuit. Despite anticoagulation therapy, clot formation is an inevitable process during ECMO, as anticoagulation only delays clotting rather than preventing it entirely. Clots can increase shear stress within the circuit, further exacerbating RBC damage. These effects are further exacerbated by the depletion of Nitric Oxide (NO) through the NO scavenging pathway: In cases of severe hemolysis, which may be induced by ECMO, excessive hemoglobin (Hb) release exhausts levels of Hb scavengers in the blood. Consequently, Hb-mediated NO scavenging takes its place, depleting NO stores. Since NO is a potent vasodilator, NO depletion can increase systemic and pulmonary vascular resistance, thrombin formation, platelet aggregation, and fibrin deposition. Additionally, the interaction between PFH and the von Willebrand factor (VWF) has been shown to promote thrombosis, further complicating the clinical course of ECMO patients [8]. The formation of thromboembolic may cause severe complications in the body, such as embolic strokes or pulmonary embolism [1].

In addition to thrombosis, ECMO-induced hemolysis can cause other severe complications. For example, excessive iron (Fe) released from hemolysis can lead to Fe overload, renal failure, and increased lung permeability. Additionally, hyperbilirubinemia has been linked with increased mortality in certain patients, such as those following cardiac bypass surgery. Excessive Red Blood Cell (RBC) damage can lead to inadequate tissue oxygenation and end-organ dysfunction or failure [1].

A recent retrospective study [9] found that VV ECMO resulted in significantly more hemolysis due to pump head thrombosis (PHT) than VA ECMO, potentially due to VV ECMO patients receiving a longer average duration of support. However, VA ECMO patients demonstrated higher overall PFH, presumably due to hemolysis from a preexisting disease [9].

## 3. Methods (Data Review)

We conducted a retrospective study of 122 patients (USF IRB # 005536) who received ECMO support at our tertiary institution between January 2020 and December 2021. We used the following inclusion criteria:

1. Age 18+.
2. Received ECMO support at our tertiary hospital between 1 January 2020 and 31 December 2021.
3. Remained on ECMO for 30 days or fewer.

Patients were categorized into three groups based on indication for ECMO support:

- COVID-19.
- Non-COVID-19 Pulmonary (usually awaiting lung transplant).
- Cardiac (with acute heart failure).

Indications for ECMO are based on the following decision-making criteria at our institution: all cases of ECMO for patients with cardiopulmonary failure follow a structured organizational framework. For cardiogenic shock, a Cardiac Shock Alert (C-Shock Alert) is activated, initiating a conference call involving a multidisciplinary C-Shock team. This team typically includes the primary cardiologist, a cardiac surgeon (if necessary), an intensivist, and sometimes an emergency room physician. During this call, the patient’s rapid clinical deterioration is assessed, and urgent consultation with a heart failure specialist is requested. Based on this discussion, the decision to proceed with ECMO is made. Subsequently, a coordinator organizes the ECMO team via text messages, ensuring proper personnel allocation and scheduling for VA-ECMO insertions in either the operating room or ICU. Following the onset of the COVID-19 pandemic, a similar structure was implemented for respiratory failure cases through a Respiratory Shock Team (R-Shock Team). This team consists of an ICU physician, admitting pulmonologist, and potentially lung transplant surgeons. After consultations cease, a final decision pathway leads to VV-ECMO initiation. Impella pumps were used in ~90% of VA patients, whereas they are rarely used for VV patients. Additionally, all VA-ECMO patients receive moderate anti-coagulation; whilst VV-ECMO rarely receives anti-coagulation. In both cases, the ECMO pump aspiration site is carefully kept below 100 mm mercury suction pressure.

Additionally, we further classified patient mortality outcomes using the following criteria.

- Expired: died during ECMO or within 30 days after discontinuation.
- Survived: survived 30 or more days after discontinuation of ECMO).

For each group and mortality outcome, we collected the following information for each patient:

- Demographics (Age, Sex).
- BMI.
- Date of ECMO Implant and Explant.
  ◦ Duration of ECMO support.
- Indication for ECMO (COVID-19, non-COVID-19 Pulmonary, or Cardiac).
  ◦ Table 1 summarizes the sample size distribution of patients by ECMO indication as well as mortality outcomes.
- Type of ECMO received (VA or VV).
- Survival Outcome (1. Expired during ECMO or within 30 days of Explant or 2. Survived 30+ days after explant).
- PFH value 24 h after ECMO initiation.
- Average PFH from the last 3 days on ECMO support.
- Average PFH from the last 5 days on ECMO support.

Significant differences were noted between the indications for ECMO among our groups.

Our findings show that for VV ECMO (including both COVID-19 and non-COVID-19 pulmonary), the most common indication was respiratory failure, followed by lung transplant. For VA ECMO, the most common indication was cardiogenic shock, followed by cardiomyopathy, cardiac surgery, and cardiac transplant. These indications align with current recommendations for VA and VV ECMO since VA ECMO is used for cardiopulmonary failure while VV ECMO is used for pulmonary failure. Previous research also supports these findings. A retrospective study with three high-volume centers showed that the most common indications for VA ECMO [10] were cardiogenic shock and cardiomyopathy. The same is true with VV ECMO, as respiratory failure remains the most common indication for the use of VV ECMO [11]. The sample sizes of indications for both VA and VV ECMO are outlined in Table 2.

We analyzed age, BMI, duration of ECMO, post 24 h PFH, last 3 day PFH, and last 5 day PFH with two-tailed *t*-tests to compare differences between Expired vs. Survived patients and, separately, COVID-19 vs. non-COVID-19 and Pulmonary vs. Cardiac patients. *T*-tests for each variable were either performed with equal variance (if the variances in the Expired and Survived groups were within a 3× multiple of each other) or unequal variance (if the difference between the Expired and Survived groups was more significant than 3×). *p* values < 0.05 indicate significance.

## 4. Results

### 4.1. Demographics: Expired vs. Survived

Patients who expired were older than those who survived for all three groups. However, this difference was only statistically significant in the COVID-19 group.

There were no significant differences in BMI between Expired and Survived patients in any of the three groups.

ECMO duration trended longer in Expired patients than in Survived patients for all three groups, but results were not statistically significant.

Complete data regarding demographic differences in Expired vs. Survived groups are reported in Table A1 in the Appendix A; individual comparisons of the three groups’ demographics are described in Section 4.2, Section 4.3 and Section 4.4.

### 4.2. Demographics: COVID-19 vs. Non-COVID-19 Pulmonary

COVID-19 patients spent a significantly longer amount of time on ECMO than non-COVID-19 pulmonary patients, regardless of mortality outcome.

Regardless of mortality outcome, age, and BMI were not significantly different between COVID-19 and non-COVID-19 pulmonary patients.

Table 3 compares demographic data for both VV-ECMO patients.

### 4.3. Demographics: COVID-19 vs. Cardiac

COVID-19 patients were significantly younger than Cardiac patients, regardless of mortality outcome.

Additionally, COVID-19 patients had over double the duration of ECMO support in comparison to Cardiac patients, regardless of mortality outcome.

BMI was not significantly different between COVID-19 and Cardiac patients, regardless of mortality outcome.

Table 4 describes these two groups.

### 4.4. Demographics: Non-COVID-19 Pulmonary vs. Cardiac

Cardiac patients tended to be older than non-COVID-19 pulmonary patients, regardless of mortality outcome. However, the results were not statistically significant.

BMI and duration of ECMO were not significantly different between Cardiac and non-COVID-19 pulmonary patients, regardless of mortality outcome.

See Table 5 for a breakdown.

### 4.5. PFH: Expired vs. Survived

PFH values over the last 3 days were significantly higher in Expired COVID-19 patients than in Survived COVID-19 patients. However, the Last 3 Day PFH values were not significantly correlated with mortality in either the Cardiac or non-COVID-19 pulmonary groups.

PFH values over the last 5 days were significantly higher in Expired COVID-19 patients than in Survived COVID-19 patients. However, the Last 5 day PFH values were not significantly correlated with mortality in either the Cardiac or non-COVID-19 pulmonary groups.

PFH values 24 h post initiation were not significantly different among Expired vs. Survived patients in any of the three groups.

See Table 6 for a breakdown.

### 4.6. Plasma-Free Hemoglobin: COVID-19 vs. Non-COVID-19 Pulmonary

Non-COVID-19 pulmonary patients had significantly higher PFH values over the last 3 days than COVID-19 patients, regardless of mortality outcome.

Last-day averages were not significantly different among non-COVID-19 pulmonary vs. COVID-19 patients, regardless of mortality outcome.

PFH values 24 h post initiation were not significantly different among non-COVID-19 pulmonary vs. COVID-19 patients, regardless of mortality outcome.

For full data regarding PFH differences in COVID-19 vs. non-COVID-19 Pulmonary Groups, see Table A2 in the Appendix A.

### 4.7. Plasma-Free Hemoglobin: COVID-19 vs. Cardiac

Cardiac patients had significantly higher PFH values over the last 3 days than COVID-19 patients, regardless of mortality outcome.

Cardiac patients also had significantly higher PFH values over the last 5 days than COVID-19 patients, regardless of mortality outcome.

PFH values 24 h post initiation were not significantly different among Cardiac vs. COVID-19 patients, regardless of mortality outcome.

For full data regarding PFH differences in COVID-19 vs. Cardiac Groups, see Table A3 in Appendix A.

### 4.8. Plasma-Free Hemoglobin: Non-COVID-19 Pulmonary vs. Cardiac

PFH values 24 h post initiation, over the last 3 days, and in the previous 5 days were not significantly different between Cardiac and Pulmonary patients, regardless of mortality outcome.

For full data regarding PFH differences in non-COVID-19 Pulmonary vs. Cardiac Groups, see Table A4 in the Appendix A.

## 5. Discussion

In our analysis, we noticed significant differences between the groups analyzed in terms of both demographic features and PFH trends.

### 5.1. Age and Mortality

Our findings suggest that elderly COVID-19 patients had worse mortality than younger patients. This aligns with previous research confirming advanced age is a significant risk factor for mortality in COVID-19 patients [12]. Age-related decline in respiratory, cardiac, and immune function and higher prevalence of comorbidities exacerbate the severity of the patient’s overall condition [12]. A retrospective cohort study by Zhou et al. [12] showed that older age in COVID-19 patients was heavily correlated (*p* < 0.0001) with worse mortality outcomes, which aligns with our findings [12].

Additionally, research by Williamson et al. [13] demonstrated that each additional decade of life increases the risk of COVID-19 mortality, with those aged over 80 years having an adjusted hazard ratio for death that is over 20 times greater than those aged 50–59 [13].

While increased age was not significantly correlated with increased mortality outcomes in the non-COVID-19 Pulmonary or Cardiac groups, a positive trend was seen. We expect that, with a larger sample size, these results will be significant due to the known fact of age as a comorbidity.

### 5.2. Duration of ECMO Support

In this study, COVID-19 patients required significantly longer ECMO support than non-COVID-19 pulmonary and cardiac patients. One proposed hypothesis for this is that the severe and prolonged course of ARDS in many COVID-19 patients necessitates a longer duration of respiratory support [14]. Extended ECMO durations can also be explained by the extensive inflammatory response during an acute COVID-19 infection (see COVID-19, hypercoagulability, and hemolysis section). Longer ECMO durations in COVID-19 patients are consistent with previous research. In a systematic review of COVID-19 and non-COVID-19 ARDS patients undergoing ECMO by Aljishi et al. [14], COVID-19 patients remained on ECMO for the past 14 days, while 20% of non-COVID-19 patients had already been weaned off. Additionally, COVID-19 patients would often require more extensive time in the intensive care unit even after weaning off ECMO.

### 5.3. PFH as a Predictor of Mortality

Previous research has described a link between PFH and mortality. While other researchers have established a connection between mortality and PFH after 24 h, PFH average, and PFH peak, our results also indicate significance in late, sustained spikes in PFH, especially in COVID-19 patients.

While elevated PFH over the last 3 days and last 5 days of ECMO support were significantly correlated with mortality in our study, values after 24 h were not significant, contradicting earlier findings by Omar et al. [1]. Although values for the Cardiac and non-COVID-19 Pulmonary groups after 24 h appeared to be potentially significant, results were skewed by outliers.

1. Cardiac Group, first 24 h:
  - While the Cardiac Survived Group seemed to have a much higher PFH in the first 24 h (217) than the Expired group (67.39), the difference was not statistically significant as the Survived group average was skewed by an outlier. For instance, one patient in the group had a PFH value of 1270 after 24 h, while the next highest PFH value for the same group was only 230. After omitting the outlier, the average drops from 217 to 100.
  - As our institution now routinely monitors PFH during ECMO as a sign of hemolysis, we hypothesize that our clinicians were more inclined to intervene and make circuit adjustments when PFH values were high, which explains the lack of PFH difference in the last 3 and 5 days for this group. Additionally, there is a possibility that these patients had a higher degree of pre-existing hemolysis as a result of chronic cardiac disease, which would manifest as high PFH values initially but normalizing values as the patient continues to stabilize under ECMO. For example, the patient with PFH of 1270, as mentioned above, quickly normalized to 330 after 24 h and near 0 towards the end of their ECMO course, which supports our hypotheses.
2. Non-COVID-19 Pulmonary, first 24 h:
  - The difference in PFH values in the non-COVID-19 pulmonary group between the Expired (49.44) and Survived (110) groups was near significant (*p* = 0.057), but did not meet the criteria for significance based on our threshold (*p* < 0.05). Similarly to the Cardiac group, this discrepancy could also be explained by some outliers in the Survived group. For example, three patients had PFH values in the 200+ range while all other patients in the group had PFH values near baseline.
  - Similarly to the Cardiac group, we also believe that a combination of pre-existing hemolysis and early clinician intervention explain the high PFH values early, with stabilizing values later in the ECMO course. For instance, all three patients with early PFH of 200+ had rapidly declining PFH values over the next several days as their condition stabilized.

We propose that this could be due to previous research by Omar et al. [1] and others demonstrating this link between PFH and mortality, healthcare professionals at our tertiary institution have become more responsive to early spikes in PFH and have made earlier modifications to the ECMO setup to alleviate further hemolysis.

Our findings highlight the potential benefits of frequent PFH monitoring in these patients. The role of PFH in promoting hemolysis and thrombosis through its interaction with NO, VWF, Fe, and other mechanisms highlights the need for careful management of coagulation parameters in ECMO patients. Reducing thrombotic complications can potentially improve the prognosis of these critically ill patients. Additionally, implementing routine monitoring of PFH levels and early intervention when levels rise above critical thresholds could be beneficial. A lack of intervention can increase the risk of complications such as renal dysfunction, disseminated intravascular coagulation (DIC), and multi-organ failure.

Our hypothesis regarding the need for frequent monitoring is further supported by a recent paper studying the effects of durations of arterio-venous shunting on blood decomposition [15]. This type of shunting is used during trial-off periods when weaning patients off ECMO support. It involves blood circulating continuously within ECMO’s artificial membranes rather than through the patient’s circulation. The researchers found that extended durations of this trial-off procedure, specifically >4 h, are associated with a substantially increased risk of circuit thrombosis, blood cell decomposition, and subsequent increase in plasma-free hemoglobin. This lends further credence to the contribution of ECMO’s artificial surfaces to thrombosis and hemolysis.

The Extracorporeal Life Support Organization (ELSO) reports safe and effective practice guidelines for ECMO support based on extensive experience, research, and consensus. ELSO supports the idea of PFH monitoring in ECMO patients. ELSO recommends holding PFH values under 10 mg/dL under most conditions. Additionally, they mention that PFH levels >50 mg/dL indicate severe hemolysis, and the cause should be investigated should values exceed this number. This proactive approach can help mitigate the adverse effects of hemolysis and improve patient outcomes [16].

### 5.4. COVID-19, Hypercoagulability, and Hemolysis

From our results, COVID-19 patients required ECMO for a longer duration and expired at lower PFH values than their non-COVID-19 Pulmonary and Cardiac counterparts. These results are supported by previous research that heavily linked COVID-19 with increased hypercoagulability, inflammation, and hemolysis.

Recent research has shown strong evidence of the presence of a hypercoagulable state in patients with COVID-19, which could predispose to increased thrombosis and hemolysis as blood traverses the ECMO circuit [17]. Previous research has established a link between COVID-19 infection and hemolysis, which may be explained by a combination of several mechanisms [18].

COVID-19 infection induces auto-antibodies against RBC membranes, weakening their structure and inducing hemolysis. Additionally, it has been proposed that SARS-CoV-2 binds to Band3 protein on the surface of RBCs. As Band3 protein is critical in docking structural proteins, maintaining membrane integrity, and oxygen release, a Band3 disturbance increases the risk of severe hemolytic effects, including hypoxia and metabolic imbalance [18].

COVID-19 infection and subsequent cytokine storm have also been shown to increase the expression of cluster of differentiation 147 (CD147), a key mediator of SARS-CoV-2 endocytosis into RBCs. This increased invasion serves as another contributing factor to hemolytic anemia in COVID-19 patients [18].

Mild hemolytic anemia has also been shown to be a single independent predictor of mortality in COVID-19 patients, according to a retrospective case–control study of over 2500 patients. Additionally, the study demonstrated a direct correlation between the degree of anemia and mortality risk. Interestingly, these trends held consistent even at milder levels of anemia, indicating that anemia can serve as an early indicator of poor survival outcomes in COVID-19 patients [19]. Hemolysis and ECMO Configuration

The significantly higher PFH values observed in cardiac patients compared to COVID-19 and non-COVID-19 pulmonary patients may be explained by the type of ECMO used. Cardiac patients typically receive Veno-Arterial ECMO (VA-ECMO), which is associated with higher rates of hemolysis due to the increased shear stress and mechanical trauma by differences in cannulation as well as the use of an artificial heart pump. This trend is supported by previous research [4]. On the other hand, COVID-19 and non-COVID-19 pulmonary patients received Veno-Venous ECMO (VV-ECMO), which historically causes less hemolysis due to the lower pressure compared to a VA system. Our findings are supported by a retrospective single-center analysis by Appelt et al. [9], who also used PFH as a marker for hemolysis and noted a greater number of instances of PFH reaching >500 mg/L in VA patients than VV patients. However, they also described that, even before ECMO initiation, PFH values were significantly greater in VA ECMO patients, suggesting that much of the difference in PFH may be due to differences in indications between VA and VV ECMO, rather than the ECMO support itself.

## 6. Limitations

Our study has several limitations.

Firstly, the apparatus used to measure PFH at our tertiary institution is not precise at PFH levels <30 mg/dL. Because of this, any PFH value labeled as “<30 mg/dL” in a patient’s lab results is charted in this report as 30 mg/dL. Replacing all “<30 mg/dL” values with 0 mg/dL did not alter any significant findings.

Additionally, our sample size was limited due to limited COVID-19 patients receiving ECMO in the years following 2021 and a new ECMO pump being implemented in 2022.

While most patients had PFH values recorded every 24 h, some values were never recorded or recorded at intervals greater than or less than precisely 24 h.

The retrospective nature of our study additionally limits us in our ability to account for certain factors:

- We could not account for pre-ECMO clinical severity scores and comorbidities, as these factors were not systemically recorded for all patients.
- As PFH monitoring during ECMO is still being studied, our institution does not routinely record Pre-ECMO PFH levels.
- Oxygenator clotting risk was primarily managed clinically, and specific parameters, including pre and post-oxygenator pressures and coagulation parameters, were not routinely recorded in the EMR.
- ECMO pump settings (flow, RPM, etc.) were not systemically documented and we were therefore unable to account for these variables.

Future, prospective studies with incorporation of these variables may be beneficial to better isolate the predictive value of PFH.

We acknowledge that bleeding events, in addition to thrombotic events, are a common and significant complication in ECMO patients. While our study focused primarily on hemolysis and PFH levels, we recognize that bleeding events can significantly impact patient outcomes and potentially interact with hemolysis. We did not systematically collect data on the incidence, severity, or location of bleeding events, nor did we analyze the relationship between bleeding and PFH levels. Future research should prospectively evaluate the association between bleeding events, PFH, anticoagulation strategies, and other coagulation parameters to gain a more comprehensive understanding of the complex interplay between hemostasis and hemolysis in ECMO patients.

Regarding statistical analysis, our study primarily aimed to identify potential associations between PFH trends and mortality across three subgroups in an exploratory way. For instance, as PFH spikes in COVID-19 patients had not been heavily studied, we prioritized sensitivity over specificity in our findings. Additionally, due to the small sample size of some of our cohorts (for instance, non-COVID-19 pulmonary survivors *n* = 10), adjustments such as Bonferroni may have increased the risk for type II errors. We acknowledge limitations in this statistical approach, and future studies would be beneficial to specifically focus on further analyzing specific trends found in our data.

## 7. Conclusions

This study highlights the predictive value of late, sustained spikes in PFH in determining mortality outcomes among COVID-19 patients receiving ECMO support. This supports previous research, which has found links between early spikes in PFH, high PFH averages, high PFH peak values, and increased mortality risk. Our findings, combined with previous studies, suggest there may be benefits in more vigilant monitoring of PFH levels, and that sustained elevations in PFH may serve as an early warning indicator for worsening patient status. This suggestion is supported by ELSO, which recommends that levels be kept under 10 mg/dL and that the causes of PFH spikes over 50 mg/dL should be investigated and corrected. Improving ECMO circuit technology and enhancing patient management protocols are essential for improved patient outcomes.

Additionally, the differences in PFH levels and duration of support between COVID-19, non-COVID-19 pulmonary, and cardiac ECMO patients point to the influence of disease course and ECMO configuration on hemolysis and mortality rates. Further research is essential to develop strategies to minimize ECMO-induced hemolysis and improve outcomes for all ECMO patients.

Overall, this summary provides valuable insight into the significant differences between ECMO subgroups and identifies PFH as a strong indicator of mortality outcomes for certain groups such as our COVID-19 cohort. This provides a framework for future research in narrowing down specific trends regarding the utility of PFH in improving patient outcomes on ECMO.

## Figures and Tables

**Table 1:** Distribution of patients receiving ECMO (*n*).

	2020		2021	
	Expired	Survived	Expired	Survived
COVID-19	10	3	26	11
non-COVID-19 Pulmonary	5	3	17	7
Cardiac	9	4	20	7
Total	24	10	63	25
Total	34		88	

**Table 2:** ECMO Indications.

VV ECMO			VA ECMO		
Indication	Sample Size (*n*)	Percentage (%)	Indication	Sample Size (*n*)	Percentage (%)
Cardiogenic shock	0	0	Cardiogenic shock	27	69.23
Cardiomyopathy	1	1.25	Cardiomyopathy	3	7.69
Cardiac arrest	2	2.5	Cardiac arrest	1	2.56
Cardiac surgery	0	0	Cardiac surgery	3	7.69
Cardiac transplant	0	0	Cardiac transplant	3	7.69
Lung transplant (awaiting)	10	12.5	Lung transplant (awaiting)	0	0
Respiratory failure—various causes	67	83.75	Respiratory failure—multiple causes	2	5.13

**Table 3:** Demographics of COVID-19 vs. non-COVID-19 Pulmonary patients.

	Age (Years)		BMI (kg/m^2^)		Days on ECMO	
	COVID	Pulmonary	COVID	Pulmonary	COVID	Pulmonary
Expired	52.29	52.5	31.17	27.73	14.47 * (0.00014)	7.18
Survived	40.86	44.3	28.28	28.92	12.42 * (0.0194)	6.2

*: significantly different.

**Table 4:** Demographics of COVID-19 vs. Cardiac patients.

	Age (Years)		BMI (kg/m^2^)		Days on ECMO	
	COVID	Cardio	COVID	Cardio	COVID	Cardio
Expired	52.29 * (0.013)	59.53	31.17	29.18	14.47 * (0.00005)	6.96
Survived	40.86 * (0.023)	53.6	28.28	28.27	12.42 * (0.004)	5.6

*: significantly different.

**Table 5:** Demographics of non-COVID-19 Pulmonary vs. Cardiac patients.

	Age (Years)		BMI (kg/m^2^)		Days on ECMO	
	Pulmonary	Cardio	Pulmonary	Cardio	Pulmonary	Cardio
Expired	52.5	59.53	27.73	29.18	7.18	6.96
Survived	44.3	53.6	28.92	28.27	6.2	5.6

**Table 6:** PFH (mg/dL) of Expired vs. Survived patients receiving ECMO.

	First 24 Hours		Last 3 Days		Last 5 Days	
	Expired	Survived	Expired	Survived	Expired	Survived
COVID-19	61.47	45	45.41 * (0.031)	35.48	46.25 * (0.038)	38.14
Pulmonary	49.44	110	65.56	72.96	62.68	68.07
Cardiac	67.39	217	60.96	61.67	66.94	67.55

*: significantly different.

## Data Availability

The original contributions presented in this study are included in the article. Further inquiries can be directed to the corresponding author.

## Appendix A

Table A1 shows our comparisons and significant differences between demographics in Expired vs. Survived patients.

**Table A1:** Demographics of Expired vs. Survived patients.

	Age (Years)		BMI (kg/m^2^)		Days on ECMO	
	Expired	Survived	Expired	Survived	Expired	Survived
COVID	52.29 * (0.00055)	40.86	31.17	28.28	14.47	12.42
Pulmonary	52.5	44.3	27.73	28.92	7.18	6.2
Cardiac	59.53	53.6	29.18	28.27	6.96	5.6

*: significantly different.

Table A2 shows our comparisons and significant differences between PFH values in COVID-19 vs. non-COVID-19 Pulmonary patients.

**Table A2:** PFH (mg/dL) of COVID-19 vs. non-COVID-19 pulmonary patients receiving ECMO.

	First 24 Hours		Last 3 Days		Last 5 Days	
	COVID	Pulmonary	COVID	Pulmonary	COVID	Pulmonary
Expired	61.47	49.44	45.41 * (0.049)	65.56	46.25	62.68
Survived	45	110	35.48 * (0.0317)	72.96	38.14	68.07

*: significantly different.

Table A3 shows our comparisons and significant differences between PFH values in COVID-19 vs. Cardiac patients.

**Table A3:** PFH (mg/dL) of COVID-19 vs. Cardiac patients receiving ECMO.

	First 24 Hours		Last 3 Days		Last 5 Days	
	COVID	Cardio	COVID	Cardio	COVID	Cardio
Expired	61.47	67.39	45.41 * (0.037)	60.96	46.25 (0.012)	66.94
Survived	45	217	35.48 * (0.018)	61.67	38.14 (0.044)	67.55

*: significantly different.

Table A4 shows our comparisons and significant differences between PFH values in non-COVID-19 Pulmonary vs. Cardiac patients.

**Table A4:** PFH (mg/dL) of non-COVID-19 pulmonary vs. Cardiac patients receiving ECMO.

	First 24 Hours		Last 3 Days		Last 5 Days	
	Pulmonary	Cardio	Pulmonary	Cardio	Pulmonary	Cardio
Expired	49.44	67.39	65.56	60.96	62.68	75.32
Survived	110	217	72.96	61.67	68.07	67.55
